# Anterior segment structure changes caused by different luminance light after implantable collamer lens surgery

**DOI:** 10.1186/s12886-023-03014-z

**Published:** 2023-06-16

**Authors:** Xia Li, Min Wang, Weiwen Dong, Jinfeng Cai

**Affiliations:** 1 Department of Ophthalmology, Shanghai Aier Eye Hospital, Shanghai, China; 2 Department of Auxiliary Examination, Shanghai Aier Eye Hospital, Shanghai, China

**Keywords:** ICL surgery, Vault, Light intensity, Pupil, Lens

## Abstract

**Backgrounds:**

To investigate the changes of anterior eye segment with implantable collamer lens (ICL) under mesopic and photopic conditions.

**Method:**

Forty-seven eyes of myopic patients who underwent ICL V4c implantation were included. Three months after surgery, the pupil diameter (PD), dynamic vault, ACD (distance from the posterior corneal surface to the anterior lens surface), ACD-ICL (distance from the posterior corneal surface to the anterior ICL surface), and anterior chamber angle parameters were measured using the anterior segment optical coherence tomography (AS-OCT, Carl Zeiss AG, Germany) under completely mesopic (0 lx) and photopic (5290 lx) lighting conditions.

**Results:**

Compared with mesopic conditions, a significant decreased vault was detected in photopic conditions (486.7 ± 186.1 μm versus 643.5 ± 191.2 μm, *p* < 0.001), while the ACD-ICL increased significantly (2.54 ± 0.24 mm versus 2.37 ± 0.23 mm, *p* < 0.001). The pupil was smaller in photopic condition (2.66 ± 0.23 mm versus 5.62 ± 0.55 mm, *p* < 0.001). ACD didn’t change(3.32 ± 0.24 mm versus 3.31 ± 0.22 mm, *p* = 0.079). The change of the vault was positively related to the changes of the PD (*r*^*2*^ = 0.301, *p* = 0.04). There were no statistical difference between the change of vault and the change of ACD-ICL (158.0 ± 58.1 μm versus 165.9 ± 65.3 μm, *p* = 0.320).

**Conclusion:**

When exposed to high intensity light after ICL surgery, the pupil constricted, vault decreased, ACA widened and ACD-ICL increased. All these changes were caused by the change of iris not the crystalline lens.

## Introduction

The implantable collamer lens (ICL) (STAAR Surgical, Monrovia, CA) has been demonstrated to be an effective and safe refractive surgery to solve the ametropia with a low complication rate and high rate of satisfaction for a wide range of ametropia [1]. One of the most important safety parameter is the vault: the distance between the posterior surface of the ICL and the anterior surface of the crystalline lens. The extreme low vault was considered to be associated with the formation of the anterior subcapsular cataract [1], and the extreme high vault was associated with pigment dispersion and narrowed anterior chamber angle and even glaucoma [2, 3].

The illuminance level was usually over 5000 lx especially in outdoor playground on sunny days [4] and decreased to 0 lx at night which was the normal experience for all the patients. When the eye exposed to light, the pupil will constrict. When the PD changes, the vault will change accordingly [5–9]. The safety vault should take into consideration of the change with the light. It is very important to make clear what the anterior segment was in different lighting conditions after ICL surgery.

When the light intensity changes, the PD changes and the position of the ICL will move backwards and forwards with the iris tension changes. Several researches found the loss of corneal endothelium cells were associated with the decreased distance between the corneal endothelium and central ICL [10–15]. The vault, the structure of the anterior chamber angle, anterior chamber depth to the crystalline lens (ACD), and anterior chamber depth to the ICL(ACD-ICL) was of paramount importance to the safety of ICL surgery. Anterior chamber depth (ACD), anterior chamber angle (ACA), scleral spur angle (SSA), angle opening distance at 500 μm (AOD500) and 750 μm (AOD750), trabecular-iris space area at 500 μm (TISA500) and 750 μm (TISA750) was the parameters frequently used evaluate the anterior segment structures.

AS-OCT is a piece of useful equipment to measure the parameters of the anterior segment. The purpose of this study is to observe the structure of the anterior segment in different lighting conditions after ICL surgery.

## Methods

### General data

The subjects were myopia patients who underwent ICL implantation in Shanghai Aier Eye hospital from December 2017 to April 2021. All the patients in this study were in accordance with the ethical standards of the the responsible national committeeand the Helsinki Declaration of 1975 and its later amendments or equivalents. The study was allowed by the Shanghai Aier Eye Hospital ethics board.

Inclusion criteria: aged 18 to 40 years old; no history of any intraocular surgery; no history of any other ocular diseases such as cataract, glaucoma, retinal diseases ,et al.; no history of general and ophthalmological medicine.

Exclusion criteria: Anterior chamber depth < 2.8 mm (Pentacam, HR; Oculus Optikgeräte GmbH, Wetzlar, Germany); corneal endothelium cells density < 2000/cm^2^(Topcon Corp, Tokyo, Japan); corneal endothelium cells dysfunction; history of fundus diseases; chronic systemic diseases such as collagen vascular diseases or diabetes mellitus.

47 right eyes from 47 patients who underwent ICL surgery were recruited in this study. All the subjects were informed of the study and signed the written consent form.

### ICL surgery

The detailed preoperative ophthalmologic examination was performed and indications were strictly evaluated before the surgery. The surgery was performed by the same surgeon. Tropicamide Phenylephrine Eye Drops (Santen Pharmaceutical Co., Ltd., Japan) were used 30 min before surgery for pupil dilation. Topical anesthesia was administered three times to the operated eye 5 min before surgery with oxybuprocaine (Santen Pharmaceutical Co., Ltd., Japan). Routine disinfection was performed. A 3.0 mm self-sealing clear corneal incision was made at 9 o’clock of corneal limbus. And a proper sized and diopter ICL was injected into the anterior chamber with an injector. Medical Sodium Hyaluronate Gel (Bausch & Lomb Incorporation, USA) was used as the viscoelastic substance. With a hook, both haptics of the ICL were carefully placed beneath the iris. After removal of the viscoelastic substance, corneal wound was closed by stromal hydration.

### Parameter measurement

The postoperative ACD, ACD-ICL, the PD on the iris plane, the parameters of the anterior chamber angle, and the dynamic vault in different lighting conditions were measured using ZEISS Visante AS-OCT (Carl Zeiss AG, Germany) 3 months after the operation. Scans were obtained in the horizontal meridian and each scan was measured three times. First, mesopic measurements were performed in a room luminance of 0 lx, which was monitored using a digital lux meter (AS803; China). And then, turn on the illuminating light, and photopic measurements were performed. The luminance of the light was 5290 lx.

### Statistical analysis

To obtain a sample size with an overall power of 80%, a paired Student’s *t-*test was conducted. Based on an effect size of 0.5, we calculated that we would need 34 eyes(G*Power Version 3.1.9.2,Franz Faul, Universitat Kiel, Germany). The normality of the data was checked by Kolmogorov-Smirnov test. Data that followed a normal distribution were presented as the mean ± SD. If not, data were presented as median (lower quartile and upper quartile). We applied the Paired Sample T test to compare ACD-ICL, ACD, postoperative vault, ACA, SSA, AOD500, AOD750, TISA500, TISA750, and pupil diameter (PD) under different lighting conditions. Pearson correlation statistical test was used to estimate the correlation between two groups. The automatic linear modeling analysis was used to predict the independent factors for PD change. All data were analyzed with SPSS software (SPSS 20.0). Differences were considered statistically significant at *P* < 0.05.

## Results

A summary of preoperative demographic characteristics of the patients were shown in Table 1. The mean age was 27.1 ± 5.5( ranged from 18 to 50). The postoperative uncorrected distance visual acuity (UDVA) was 1.0 ± 0.2, and the spherical equivalent (SE) was + 0.25D ± 0.5D.

**Table 1:** The preoperative parameters of patients

	Mean(x± s)	Range
ACD(mm)	3.32 ± 0.28	2.8 to 3.85
ICL size(mm)	12.71 ± 0.31	12.1 to 13.7
WTW(mm)	11.45 ± 1.25	11.1 to 12.47
Spherical diopter (D)	−8.17 ± 4.33	−4.75 to−12.5
Cylindrical diopter (D)	−1.3 ± 0.4	−3.75 to 0

ACD: anterior chamber depth, WTW: white to white

Of the 47 ICLs, 3 (6.4%) was 12.1 mm, 29(61.7%) was 12.6 mm, and 15(31.9%) was 13.2 mm. Twenty-eight (59.57%) was torci ICL. The power of the Toric ICL was − 11.41 ± 2.74DS, 1.91 ± 0.88DC. 19(40.43%) was ICL. The power of the ICL was − 9.79 ± 2.42DS.

The postoperative PD, Vault, ACD, ACD-ICL, ACA, SSA, AOD500, AOD750, TISA500, TISA750 in different lighting conditions was shown in Table 2. As expected, the PD became much smaller (2.66 ± 0.23 mm versus 5.62 ± 0.55 mm ,*p* < 0.001) when the luminance level increased from completely dark (0 lx) to 5290 lx. The ACD had no statistical difference in mesopic and photopic conditions while the vault, ACA, SSA, AOD 500, AOD 750, TISA 500, TISA 750 decreased in photopic condition than that in mesopic condition.

**Table 2:** Postoperative pupile size, vault, anterior segment structure Changes under different lighting conditions

	Mesopic	Photopic	Change mean ± SD range	*t*	*P* ^*^
PD(mm)	5.62 ± 0.55	2.66 ± 0.23	3.01 ± 0.57 2.00 to 4.50	38.129	< 0.001
Vault(um)	643.5 ± 191.2	486.7 ± 186.1	158.7 ± 57.66 30 to 300	18.515	< 0.001
ACD(mm)	3.32 ± 0.24	3.31 ± 0.22	0.01 ± 0.03 -0.08 to 0.07	1.797	0.079
ACD-ICL (mm)	2.37 ± 0.23	2.54 ± 0.24	-0.16 ± 0.07 -0.36 to -0.06	-19.138	< 0.001
ACA (°)	15.9 ± 3.8	20.5 ± 3.41	-4.6 ± 2.5 1.5 to 11.0	-10.319	< 0.001
SSA(°)	32.7 ± 7.3	42.2 ± 5.7	-9.5 ± 6.4 -0.5 to -27.4	-8.481	< 0.001
AOD500 (mm)	0.331 ± 0.091	0.463 ± 0.085	-0.132±-0.088 -0.01 to -0.39	-8.676	< 0.001
AOD750 (mm)	0.444 ± 0.117	0.582 ± 0.105	-0.138±-0.102 -0.07 to -0.38	-7.79	< 0.001
TISA500 (mm^2^ )	0.119 ± 0.036	0.166 ± 0.031	-0.047 ± 0.033 -0.15 to 0.01	-8.095	< 0.001
TISA750 (mm^2^ )	0.215 ± 0.058	0.295 ± 0.052	-0.080 ± 0.055 -0.25 to 0	-8.438	< 0.001

^*^Paired sample test

PD: pupil diameter, ACD: anterior chamber depth, ACD-ICL: anterior chamber depth to the anterior surface of the ICL, ACA: anterior chamber angle, SSA: scleral spur angle, AOD500: angle opening distance at 500 μm, AOD750: angle opening distance at 750 μm, TISA500: trabecular-iris space area at 500 μm, TISA750: trabecular-iris space area at 750 μm

The decrease of the vault in photopic condition was 158.7 ± 57.66 μm, and the increase of ACD-ICL was 0.16 ± 0.07 mm( 160 ± 70 μm). Comparing the values with Paired Samples T Test, there were no statistical difference between them( *t* = -1.005, *p* = 0.320).

**Fig. 1:**
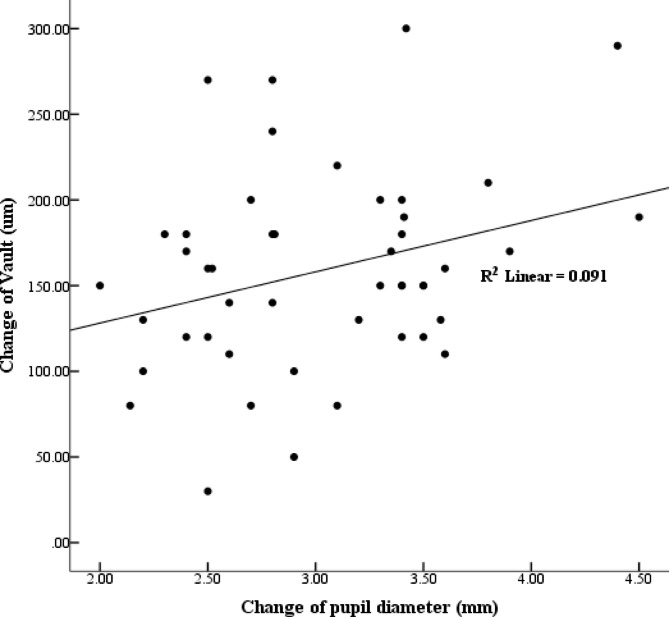
The relationship between the vault change relative to change of PD under different lighting conditions

Figure 1 showed the relationship between the vault change relative to change of PD under different lighting conditions. The decrease of the vault in photopic conditions was positively correlated with the changes of PD(*r*^*2*^ = 0.301, *p* = 0.04). The change of PD was positively correlated with the PD in mesopic condition (*r*^*2*^ = 0.922, *p* < 0.001) and negatively correlated with the PD in photopic condition(*r*^*2*^ = -0.403, *p* = 0.004). Linear modeling analysis was performed, and PD in mesopic condition was the independent factor for the PD change.

## Disccusion

In this study, when the illuminance light changed, the average change of the vault was 158.7 ± 57.66 μm, from 643.5 ± 191.2 μm in mesopic condition (0 lx) to 486.7 ± 186.1 μm (5290 lx). Kato [8] reported the vault to decrease from 521.1 ± 220.4 μm in scotopic conditions(4 lx) to 476.1 ± 219.6 μm in photopic conditions(400 lx). Xiong et al. [16] also reported the vault decreased 116 ± 66 μm from mesopic condition(0.11 lx) to photopic condition(5962 lx). Gonzalez-Lopez et al. [5] reported that the vault decreased 167 ± 70, from 540 ± 252 μm in scotopic conditions(0.5 lx) to 374 ± 208 μm in photopic conditions(18,500 lx).

The difference could be ascribed to the different lighting conditions and the following difference of PD. The change of vault in this study was 158.7 ± 57.66 um which was close to the results of Gonzalez-Lopez(167 ± 70um) [5]. The PD was 5.62 ± 0.55mm and 2.66 ± 0.23mm in mesopic and photopic conditions respectively. The mean change in this study was 3.01 ± 0.57mm which was also comparable to the results of Gonzalez-Lopez et al. [5] (3.10 ± 0.70mm). While the change of PD found in Xiong’ study [16] was 1.91 ± 0.70 mm, and from 4.91 ± 0.75 mm to 3.64 ± 0.77 mm in Kato’s study [8], which was smaller than that of the present study and Gonzalez-Lopez’s study. A smaller change of PD corresponded to smaller change of vault. When induced by stronger light, the pupil constricted more significantly which would induce a stronger iris tension. The ICL would be pushed downward more towards the lens which resulted in larger vault change.

When the change of the PD was analyzed, it was found that the change was positively correlated with the PD in mesopic condition and negatively correlated with PD in photopic conditions. The Automatic Linear Modeling showed the PD in mesopic condition was the independent factor of the PD change, which meant a larger PD in mesopic condition predicted a larger PD change in photopic conditions and then a larger vault change, while the PD in photopic conditions had no effect on the PD change. It could be inferred that when exposed to light (5962 lx), the PD almost reached the same level and in the completely dark(0 lx), the PD was a specific characteristic for the individual and in such circumstances, the iris still compressed the ICL.

The ACD wasn’t found any change from mesopic condition (0 lx) to photopic condition (5290 lx) in this study. It was consistent with many other previous researches [5, 8, 16]. Gonzalez-Lopez et al. [5] found a significant rise of the crystalline lens rise(CLR). Theoretically, a shallower ACD should be achieved, that was not the case as what they found in factual measurement. They explained the difference by a longer angle-to-angle(ATA) distance in photopic condition, which they deemed compensated the change of the CLR and the final ACD does not vary significantly. Reviewing the article carefully, we preferred that the crystalline didn’t change in different luminance conditions. The change of CLR should be attributed to the downward movement of the baseline of ATA, because the chamber angle opened more in the photopic condition than that in the scoptic condition. Kato S [8] also demonstrated that the lens thickness didn’t change in different luminance conditions. Therefore, we inclined to believe that the change of anterior segment structure in different luminance conditions didn’t involve the change of crystalline, which meant the crystalline didn’t change its position and form to the different lighting conditions.

There were no statistical difference between the change of the vault and the change of ACD-ICL. The decrease of the vault in photopic condition could totally attributed to the change of the ICL not the change of the crystalline lens. In other words, the change of the light intensity couldn’t induce the accomodative response of the crystalline lens. While when the accommodative response occurred, the vault decreased, and the decrease of the vault could be attributed to the backward movement of the ICL and thickness of the crystalline lens [7].

The ACA, SSA, AOD 500, AOD 750, TISA 500, TISA 750 became smaller in the mesopic condition. The changes could partly be attributed to the thickened iris root and partly should be attributed to the ICL movement towards the cornea in the mesopic condition. In this study, the ACA was only 7.9 in one patient, whose vault in mesopic condition was 940 μm and 750 μm in photopic condition. In the 3 months follow-up period, the intraocular pressure was within normal range, and there were no other complications at all. Therefore, the patient was still under close follow-up.

According to the information of the STARR cooperation, the height of the ICL ranges from 120 to 180 μm, with average height 150 μm. The change of the vault of 16 out of 49 patients was not less than 180 μm. It meant that the decrease of vault could be partly attributed to the backward movement towards the lens in some patients. Zhang x et al. [17] analyzed the position of ICL footplates using full-scale ultrasound biomicroscopy, and found that only 21.6% (29 out of 134 eyes ) was in ciliary sulcus, 2.2% (3 out of 134 eyes) was on the top of the ciliary sulcus, and the remaining 76.1% was under the ciliary sulus. Shi M [18] also reported the position of footplates of the ICL: in the ciliary processes, in the ciliary sulcus, in the back of iris or located asymmetrically for the footplates. There was a relatively wide space and the structures within the space. There are ciliary protrude, zonule there. The structures are relatively elastic and can be transformed temporarily and allows the backward and forward movement of the ICL.

One limitation of this study was that the posterior structures behind the iris were not detected with the AS-OCT, therefore, it was still a problem to answer whether the decrease of vault was associated with the transform of ICL or the movement towards to the lens, or both. Another limitation was that in our study, both eyes had underwent the surgery, however, we only chose the right eye to observe and analyzed to avoid the correlation interference. It would be better to choose alternatively right and left eyes from these patients by random.

## Conclusions

When exposed to light from mesopic condition, the changes of the anterior segment structure after ICL surgery included the constricted pupil, decreased vault, widened ACA and increased ACD-ICL. All the changes were caused by the changes of the iris. The crystalline lens didn’t change in different luminance conditions, which was verified by the unchanged ACD and the equal change of the ACD-ICL and vault.

## Data Availability

The datasets used and/or analysed during the current study are available from the corresponding author on reasonable request.

## Abbreviations

ICL: Implantable collamer lens
PD: Pupil diameter
ACD: Anterior chamber depth
ACA: Anterior chamber angle
SSA: Scleral spur angle
AOD500: Angle opening distance at 500 μm
AOD750: Angle opening distance at 750 μm
TISA500: Trabecular-iris space area at 500 μm
TISA750: Trabecular-iris space area at 750 μm
UDVA: Uncorrected distance visual acuity
SE: Spherical equivalent
CLR: Crystallline lens rise
ATA: Angle-to-angle
